# Multiple Ways to Keep FFAT Under Control!

**DOI:** 10.1177/25152564221101219

**Published:** 2022-05-11

**Authors:** Suzan Kors, Michael Schrader, Joseph L. Costello

**Affiliations:** 1 College of Life and Environmental Sciences, Biosciences, 151673 University of Exeter, Exeter, Devon, UK

**Keywords:** ACBD5, VAPB, PTPIP51, STARD3, GSK3-β, peroxisomes, ER, mitochondria, membrane contact sites

## Abstract

Peroxisomes and the ER are closely inter-connected organelles, which collaborate in the metabolism of lipids. In a recent research paper in the Journal of Cell Biology, we describe a novel mechanism by which peroxisome-ER membrane contact sites are regulated, via phosphorylation of the peroxisomal protein ACBD5. We found that the interaction between ACBD5 and the ER protein VAPB, which we have previously shown to form a tether complex at peroxisome-ER contacts, is controlled by phosphorylation of ACBD5 at two different sites of its FFAT motif – the VAPB binding site. We also identify the kinase GSK3-β as being responsible for direct phosphorylation of ACBD5 to negatively regulate interaction with VAPB, leading to reduced peroxisome-ER contacts. In this article we provide additional insights into how this work, in combination with other studies on phosphorylation of VAP interactors, suggests a complex system of both positive and negative regulation of the FFAT motif via phosphorylation.

Interactions between organelles at membrane contact sites allows the coordination of multiple cellular events, including lipid trafficking, metabolic signalling and organelle biogenesis. Contact sites are formed by the action of tethering factors on apposing organelle membranes. This generates specific exchange hubs, which allow direct transfer of lipids and other metabolites. The last decade has seen an explosion in the functional characterisation of contact sites, with multiple different tethering complexes identified for almost all types of organelle interactions (Prinz et al., 2020). However, despite clear evidence that individual contact sites are formed dynamically, how specific contacts are regulated under different physiological conditions is still mostly unknown (Valm et al., 2017). One key player in contact sites involving the ER are the Vesicle-associated membrane protein (VAMP)-associated proteins (VAPs). VAPs are ER membrane proteins, which have the capacity to mediate interactions between virtually every other organelle and the ER, via binding to proteins containing a FFAT (two phenylalanines in an acidic tract) motif. Interactions with FFAT motif-containing proteins on other organelles can provide physical tethering capacity and can also position specific complexes at the organelle interface to facilitate exchange events. FFAT motif-containing proteins include peroxisomal ACBD5, mitochondrial PTPIP51, and endosomal STARD3, amongst others (Costello et al., 2017; Di Mattia et al., 2020; Hua et al., 2017; Stoica et al., 2014). Based on VAP's importance at membrane contact sites, it can be appropriately considered to be a Versatile Access Point for other organelles to the ER membrane (Slee & Levine, 2019).

In a recent paper published in the Journal of Cell Biology we explored regulatory mechanisms that can control peroxisome-ER interactions via phosphorylation of the tether protein ACBD5 (Kors et al., 2022). Here, we summarise this work and discuss how this fits alongside other data on phosphorylation of VAP interactors to give new insights into the control of membrane contact sites involving the ER and other organelles.

Previous work has shown that peroxisome-ER contacts are significantly reduced, but not completely abolished, in cells lacking the ACBD5-VAPB tether. When ACBD5 is ablated both the number of peroxisomes, and the extent of the peroxisomal surface, in contact with the ER are reduced (Costello et al., 2017). Our new study revealed that the interaction between ACBD5 and VAPB can be regulated by phosphorylation of ACBD5 at two different sites. Both sites are within the FFAT motif, the VAPB interacting region of ACBD5, but have opposite effects. Phosphorylation of three serines in the acidic tract of the FFAT motif can increase VAPB interaction whilst phosphorylation of a single serine in position 5 of the core FFAT motif (Serine 269) reduces VAPB interaction (Figure 1). We showed that mutation of serines in the FFAT motif of ACBD5, which led to reduced VAPB interaction also reduced peroxisome-ER contacts in a complementation assay, demonstrating that phosphorylation of ACBD5 is a regulatory mechanism to control interactions between the two organelles. Furthermore, we also identified that the kinase GSK3-β interacts with the ACBD5-VAPB complex and can phosphorylate ACBD5 at Serine 269 – leading to reduced ACBD5-VAPB interactions and reducing the extent of peroxisome-ER contacts.

**Figure 1.**
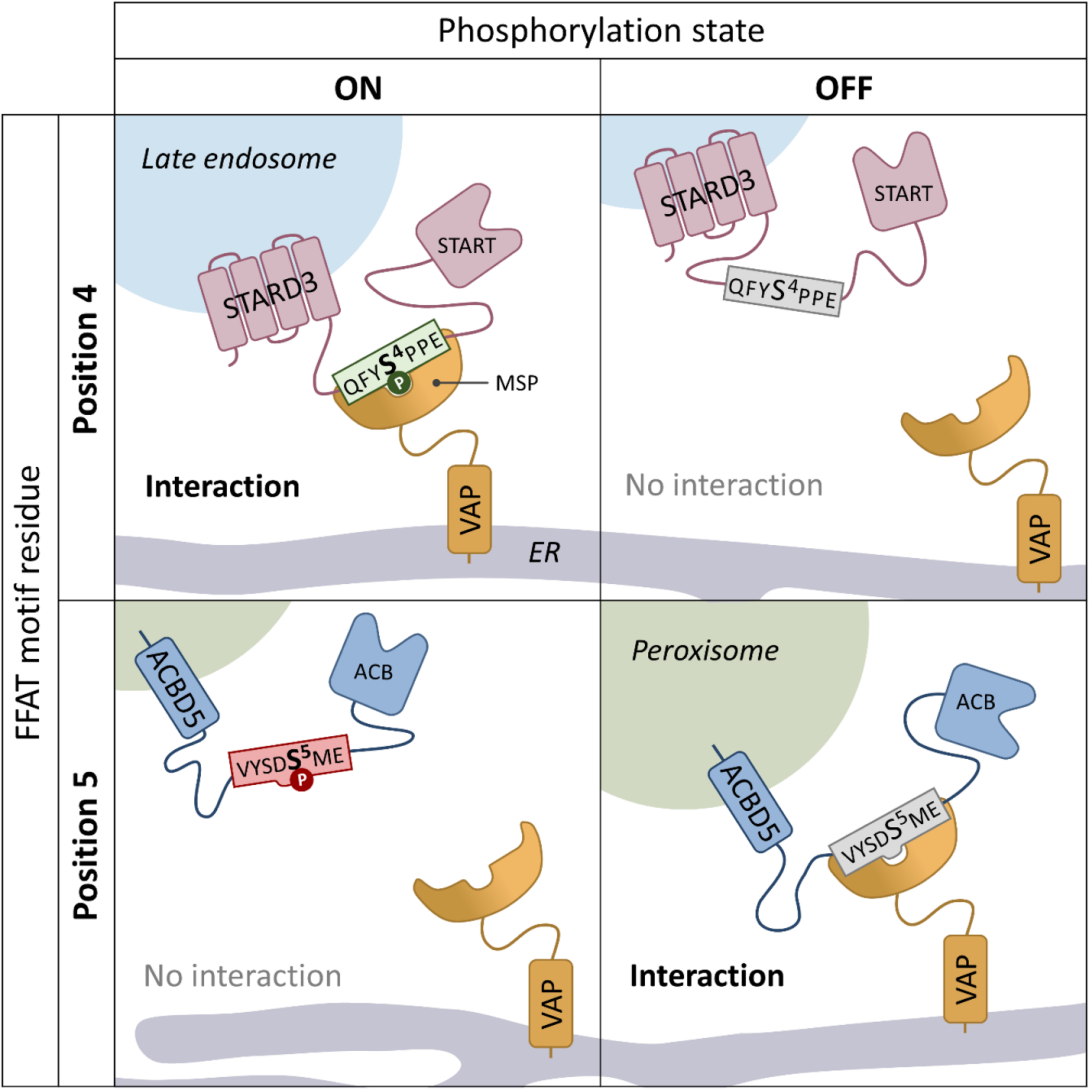
Model showing how differential phosphorylation of FFAT motif containing proteins can regulate VAP interaction via either a positive (FFAT motif phosphorylation at position 4), or negative (FFAT motif phosphorylation at position 5) mechanism.

The originally identified FFAT motif consists of seven core residues with the general consensus E^1^F^2^F^3^D^4^A^5^x^6^E^7^ surrounded by a less well defined flanking region of acidic residues (Mikitova & Levine, 2012). Based on NMR data, a two-step binding mechanism was proposed for FFAT-VAP interactions. In this model the core residues of the FFAT motif are involved in specific interactions with residues in the MSP (Major Sperm Protein) domain of VAP whilst the acidic tract mediates an initial, non-specific binding event with an electropositive surface of the MSP domain (Furuita et al., 2010).

One of the original analyses of the key elements of the FFAT motif utilised human peptides expressed in yeast with ER localisation as a proxy for VAP interaction (Mikitova & Levine, 2012). This study suggested that potential phosphorylation of a serine/threonine at position 4 would lead to increased VAP binding whilst phosphorylation at position 5 would lead to decreased VAP binding, allowing both positive and negative forms of phospho-regulation. This analysis fits with structural data which indicates that position 5 of the FFAT core would be positioned within a hydrophobic pocket in the MSP domain of VAP (Kaiser et al., 2005). Therefore, phosphorylation at position 5 could cause steric hindrance (Furuita et al., 2010). Based on this and other work, the Levine group developed prediction algorithms to score FFAT motifs, allowing many other proteins which contain FFAT motifs to be identified in a range of different organisms (Slee & Levine, 2019). In combination, our study and recent work from the Alpy group have now provided experimental validation for both positive and negative phospho-regulation of the FFAT-VAP interaction, via phosphorylation at positions 4 and 5 using the endosomal protein STARD3 and peroxisomal protein ACBD5, respectively (Di Mattia et al., 2020; Kors et al., 2022). In both studies, non-phosphorylatable and phosphomimetic mutants showed the expected alterations to FFAT-VAPB binding. Whilst *in vitro* binding studies with phospho-peptides supported the observation that phosphorylation of STARD3 at serine 4 (S209) in the FFAT motif is required for VAP interaction, our study utilised a phospho-specific antibody to S269 (FFAT core position 5) in ACBD5 to show that the population of ACBD5 which is phosphorylated at S269 does not interact with VAPB (Figure 1).

Using a modified version of the original FFAT scoring algorithm, the Alpy group identified 110 known VAP-interacting human proteins, which contain potential phosphorylation sites at position 4 of the predicted FFAT core, which they dubbed ‘Phospho-FFAT proteins’ (Di Mattia et al., 2020). Our less sophisticated analysis identified at least 11 human proteins with conventional predicted FFAT motifs, which contain a conserved serine/threonine at position 5. Interestingly, four out of the 11 proteins we identified possess FFAT motifs with serine/threonine residues at both positions 4 and 5 (ATG2B, SNX2, CALCOC1 and CERT). Whilst, of the 110 Phospho-FFAT proteins identified by Di Mattia and colleagues, 28 contain potential phosphosites at both positions 4 and 5. Structural data on the STARD3 phospho-FFAT motif showed an alteration in the structure of the FFAT-VAP complex depending on whether a phospho-serine or an aspartate residue was present at position 4. A phospho-serine at position 4 was found in a slightly different orientation compared with an aspartate, allowing stronger contact with side-chains of VAP residues. This also resulted in differences in how the downstream residues at positions 5 and 6 interacted with the hydrophobic pocket of VAP. Speculatively, this may imply that phosphorylation at position 4 could potentially alter the impact of phosphorylation at position 5. Overall, this suggests the possibility for a complex system of regulation involving both activation and inactivation of FFAT motifs depending on the phosphorylation status of the FFAT motif core (Figure 1).

As phosphorylation of serine and threonine residues results in a net gain of negative charge, phosphorylation of the acidic tract of the FFAT motif would effectively result in a more negatively charged region, and this appears to increase VAP interaction. This would be in line with a role for the acidic tract in the first step of VAP interaction and a previous study identified a phosphorylation site in the acidic tract of the FFAT-motif containing protein CERT, which enhanced VAPA binding (Kumagai et al., 2014). Both our study and that of Di Mattia and colleagues highlighted the role of phosphorylation of the acidic tract as a potential fine-tuning mechanism, which may differ from the on/off switch provided by phosphorylation of serines 4/5 in the core. As FFAT motifs are capable of competing with each other (Mikitova & Levine, 2012), such small changes in VAP affinity may allow prioritisation of specific cellular events by permitting direct access to the ER via VAP. This would also mean that protein sequences significantly different from the conventional FFAT motif, containing neither an acidic tract, nor phenylalanines or specific acidic residues in the core, could also effectively bind to VAP when phosphorylated – further complicating their identification.

Our study identified GSK3-β as the potential kinase for phosphorylation of ACBD5 at Serine 269, therefore acting as a negative modulator of ACBD5-VAPB interactions and peroxisome-ER contacts. Previous work from the Miller group has shown increased GSK3-β activity also reduced VAPB and PTPIP51 binding, controlling mitochondria-ER contact sites (Stoica et al., 2014). The nature of the phosphorylation event has not yet been elucidated but as PTPIP51 does not contain a phosphorylatable residue at position 5 in the FFAT motif, this regulation is presumably via a different mechanism. PTPIP51 does contain a threonine at position 4, phosphorylation of which has been shown to increase binding of the FFAT motif to VAP (Di Mattia et al., 2020) as well as a high number of potential phosphosites in the acidic tract. However, phosphorylation at these sites positively regulate VAPB interaction, suggesting that the role of GSK3-β in regulating the PTPIP51-VAPB interaction may be indirect. One possibility would be that GSK3-β acts indirectly by activation of a phosphatase, which could then dephosphorylate PTPIP51 at position 4, thereby leading to reduced VAPB interaction. Intriguingly, these studies suggest the potential for co-regulation of both mitochondria and peroxisome interactions with the ER via GSK3-β activity.

Disease causative mutations in VAPB, which have been suggested to alter VAPB's ability to mediate ER-organelle contacts, have been identified in patients with amyotrophic lateral sclerosis (ALS) type 8. In addition, GSK3-β can be activated by TDP-43, a protein which has previously been pathologically linked with ALS (Stoica et al., 2014). This suggests that both peroxisome-ER and mitochondria-ER connections would be altered in VAPB/TDP-43 related ALS conditions. The contribution which altered contacts make to ALS pathophysiology remains unclear, but the concept of modulating organelle interactions as a potential therapeutic approach has recently been explored in a Parkinson's disease model (Lee et al., 2018).

Overall, our study adds to the understanding of how contacts between the ER and other organelles can be regulated and also provides one of the first examples of how peroxisomal function can be regulated by phosphorylation in mammalian cells. In addition, the observation that GSK3-β activity can alter both peroxisome-ER and mitochondria-ER contacts suggest a possible co-regulation of these organelle contacts. Future work may focus on characterising the contribution of ER-organelle contacts to ALS and how modulation of these contacts, potentially via GSK3-β activity, could be used as a therapeutic strategy.
